# Men’s gender role and attitude toward sexual autonomy of women in India

**DOI:** 10.1371/journal.pone.0317301

**Published:** 2025-01-08

**Authors:** Manas Ranjan Pradhan, Prasenjit De

**Affiliations:** Department of Fertility and Social Demography, International Institute for Population Sciences (IIPS), Mumbai, Maharashtra, India; India Health Action Trust (IHAT), Uttar Pradesh Technical Support Unit (UP-TSU), INDIA

## Abstract

**Objective:**

Sexual autonomy is essential to women’s empowerment and crucial to human rights. Measurement of women’s sexual autonomy from men’s perspective is rare in India, though critical for achieving the sexual and reproductive rights of women who continue to exhibit poor sexual and reproductive health (SRH) outcomes. The study assesses Indian men’s attitudes toward women’s sexual autonomy and associated factors using a nationally representative sample of men.

**Methods:**

This study involved a total sample of 101,839 men aged 15–54 covered in the fifth round of the National Family Health Survey (2019–21). Descriptive statistics, bivariate analysis, and binary logistic regression were used to determine predictors of men’s favorable attitudes toward the sexual autonomy of women. All the statistical analyses were performed using Stata with a 5% significance level.

**Results:**

Sixty-three percent of men hold a favorable attitude toward women’s sexual autonomy. Men’s support for women’s sexual autonomy was positively correlated with their egalitarian views on household decision-making (AOR: 1.45; CI: 1.41–1.49), higher educational attainment (AOR: 1.34; CI:1.20–1.50), currently married status, media exposure (AOR: 1.17; CI: 1.12–1.21), currently working status, and wealthier household strata (AOR: 1.17; CI: 1.11–1.23).

**Conclusion:**

Results suggest promoting gender egalitarian norms through educational campaigns, community workshops held by local leaders, grassroots healthcare professionals, and non-governmental organizations, and broadening existing SRH strategies by including younger, non-literates, unmarried, unemployed, and rural men.

## Introduction

Sexual autonomy is an essential component of human rights [1] that underscores the importance of achieving sexual well-being through a pleasurable, fulfilling, and safe sexual life devoid of coercion, exploitation, discrimination, and violence [2]. The concept of autonomy is highly relevant in the context of sexual relationships [3]. Autonomy is the feeling that one’s actions stem from within oneself rather than external influences and an inner consistency between one’s desires and choices [4]. Sexual autonomy is the ability to protect and maintain informed decisions about one’s body, one’s sexuality, one’s desires, and one’s sexual experience [5, 6]. It involves making independent decisions regarding sex matters and associated activities [7]. Sexual assertiveness and perceived control are vital components of fostering healthy sexual relationships [3].

Sexual autonomy is essential to women’s empowerment [8]. Women’s sexual autonomy encompasses participation in decisions related to when, with whom, and how sexual relations are practiced, emphasizing their freedom to choose within and outside marital unions [9]. In the public health domain, scholarly articles define women’s sexual autonomy in terms of women’s ability to refuse risky and non-risky sexual activities, negotiate for safer sexual practices, and feel justified in asking a partner to use a condom [7, 8, 10, 11]. Literature suggests that women with high sexual autonomy have a significantly lower risk of experiencing adverse sexual and reproductive health outcomes, including unintended pregnancy [12], and elevate the use of modern contraceptives [13]. Women’s sexual autonomy has also been associated with a high risk of intimate partner violence [7]. With autonomy, women fight for their sexual rights, which challenge the patriarchal norms that approve men’s authority over their spouses, resulting in violent behavior from men to counter such challenges.

Individual intimate relationships and sexual interactions are impersonated by prevailing cultural norms and ideologies concerning gender and sexuality [14]. Traditional normative constructs related to masculinity, femininity, and wider gender disparities shape the roles and power dynamics within sexual relationships [15]. In many societies, the prevailing sexual double standards are one of the critical indicators of decision-making power within sexual unions [16]. Sexual double standard ideology paradoxically portrayed women as sexually weak and their sexuality placed partly or entirely under the control of their husbands or male family members [17]. It further manifests that women are protectors of the moral order and have greater control over their sexual impulses, while male sexual desires are powerful and uncontrollable for which men are not responsible [18]. Due to these cultural norms and attitudes towards female sexuality, women face significant barriers in terms of executing direct communication about their sexual needs and desires [16] and experience a lack of agency to negotiate safe sex, notably in the context of refusing sexual activity against their will [19]. In contrast, this ideology stereotypically portrays men as sexually assertive and affords them greater sexual freedom within marital relationships compared to women [20, 21]. In male-dominated societies like India, women may feel compelled to prioritize their husbands’ desires over their own, even in matters of sexual consent, due to deeply ingrained beliefs about a wife’s duty to ensure her husband’s happiness [22].

Sexual scripting theory [23] and the theory of gender and power [24] provide theoretical background for understanding men’s attitudes toward women’s sexuality. Scripting theory suggests that sexual scripts operate at three levels: cultural (institutional arrangements and collective meanings), interpersonal (interactions in specific contexts), and intrapsychic (desires and beliefs). Cultural-level scripts pose normative characteristics, compelling individuals to adhere to certain social expectations with potential repercussions for resisting them [25]. Nevertheless, individuals may adopt different scripts than those dominant at the cultural level [26]. Women are expected to conform to gender-specific scripts within a relationship characterized by stability, monogamy, and passiveness [23]. Sexually passive women may be reluctant to abdicate sexual activities that go against their partner’s desire [5]. The core theme of the theory of gender and power revolves around the proposition that sexual behavior, negotiation, and practices are a consequence of imbalanced power dynamics inherent in a patriarchal social structure power [24]. Rigid gender norms lead to social practices such as unequal power distribution within intimate relationships, acceptance of male sexual promiscuity, restrictive women’s movement, and women’s submission to their husband’s sexual desire, which further forces women to engage in risky sexual practices [19]. Due to the unequal power dynamics within a male-dominated society, women may cease resisting sexual harassment or grow accustomed to being abused by their husbands [27].

Traditional gender roles can be a barrier to women’s sexual autonomy and reduce satisfaction, discourage the use of contraceptives, and raise the risk of sexual violence [28, 29]. On the contrary, egalitarian values based on the equality principle treat all humans equally [29] and counter traditional gender roles by increasing the personal responsibility to spread positive sexual ideas and experiences [30]. Besides power dynamics and gender roles, other risk factors such as the age of the individual [31], ethnicity, race, social class, and religion [32, 33] also significantly influence men’s attitudes and behavior related to women’s sexuality. Older respondents may be less likely to adhere to traditional sexual norms as they tend to establish their own rules for sexual interaction [31]. Again, education, place of residence, acceptance of wife-beating, access to resources, and attitudes toward household decision-making influence or moderate the patriarchal construct of women’s subjugation [8, 19].

Patriarchal ideology in India is formalized through laws, customs, and rituals, as evidenced by household power relations [34]. For instance, most households in India are headed by a male member, with only 17% having a female headship [35]. A recent study found that men’s justification for wife-beating on the grounds of the wife’s refusal of sexual activity has increased from 2005–08 to 2019–21, which further shows that Indian men are unwilling to relinquish their traditional sexual scripts [36]. Sexual interaction stands out as one of the most potent arenas where both men and women experience pressure to enact gender roles [3]. Therefore, understanding the underlying attitude related to gender and sexuality is essential as it helps to comprehend awareness related to gender stereotypes and facilitates healthy interpersonal relationships. However, the scientific literature is dominated by studies that measure women’s sexual autonomy and their negotiating power to engage in safer sexual practices from women’s perspectives on men’s behavior and attitudes [8, 10, 11, 20–22, 37]. Measurement of women’s sexual autonomy directly from men’s perspective by using nationally representative data is rare. Moreover, with the ever-changing influences of urbanization, globalization, and digital media culture, how Indian men view women’s sexual autonomy needs to be explored in detail. Although traditional gender roles have historically constrained women’s autonomy, positive shifts are emerging in India, driven by urbanization, globalization, education, and digital media. Studies have found that Indian men’s overall justification for wife-beating has decreased considerably over the years [36]; nearly 83% of women can negotiate with their husbands for safer sexual practices [22], indicating a positive shift toward a gender-egalitarian view. Given the significant role of gender norms in sexual relationships, this study hypothesized that traditional authoritarian gender norms correlate with restrictive views on women’s sexual autonomy. Furthermore, higher levels of education and resources, exposure to urbanization, and digital media among men are associated with supportive attitudes toward women’s sexual autonomy. Against this backdrop, this study sought to fill the research gap by investigating men’s attitudes toward women’s sexual autonomy and associated factors using a nationally representative survey of India.

## Materials and methods

### Data source

The study used data from the fifth round of the National Family Health Survey- NFHS-5 (2019–21). The NFHS is a large-scale, multi-round survey conducted in a representative sample of households throughout India. It gathered information on various health indicators from women aged 15–49 and men aged 15–54, including attitudes toward gender roles. Informed consent procedures were followed, and only those who agreed voluntarily were interviewed by trained research investigators through Computer Assisted Personal Interview (CAPI). The round-specific survey reports include a minute description of the study design, sampling design, technique, frame, and non-response rate [35]. The present study utilized data from men’s files of NFHS-5. This study involved a total sample of 101,839 men aged 15–54 years.

### Outcome variable

The primary outcome variable was men’s attitudes toward the sexual autonomy of women. In the NFHS, men were asked if they think a wife is justified in refusing sex with her husband if she knows- a) he has a sexually transmitted disease, b) he has sex with other women, and c) if she is tired or not in mood. Furthermore, men were asked if they think that the wife is justified in asking the husband to use a condom during intercourse if she knows her husband has a sexually transmitted disease. The sexual autonomy of women was generated by combining the responses made by men. Men who responded ‘yes’ to all four circumstances were in favor of the sexual autonomy of women and coded as ‘1’. All other men were considered not to be in favor of the sexual autonomy of women and coded as ‘0’.

### Predictors

Individual, household, and community-level predictors, which could potentially influence the outcome variable, were included in the analysis. Especially, the predictors for the present study were selected based on the literature on the specific domain, and availability of data in the NFHS survey. These were age group (15–24, 25–34, 35–44, 45–54); educational attainment (no education, primary, secondary, higher); marital status (never married, currently married, others [widowed, divorced no longer living together/separated]); exposure to mass media (yes, if reads newspaper and/or magazines, listens to the radio, and watches television at least once a week or almost every day, no otherwise); occupation (not working, white-collar [professional/managerial/technical/clerical/sales], agricultural/household/domestic services, manual labor, other); caste (General, Scheduled Caste [SC], Scheduled Tribe [ST], Other Backward Classes [OBC]); religion (Hindu, Muslim, Christian, others [Sikh, Buddhist, Jain, Jewish, no religion, and others]); family history of Intimate Partner Violence (individuals were asked to answer the question of whether an individual’s father ever beat his mother, responses were ‘yes’ and ‘no’); family structure (nuclear, non-nuclear); wealth index (poorest, poorer, middle, rich, richest [already given in the NFHS dataset]); place of residence (Urban, rural); and region (North, Central, East, North-East, West, South).

Additionally, men’s attitudes toward household decision-making (egalitarian, authoritarian) and men’s justification of wife-beating (no, yes) were included as predictors. In the NFHS, men were asked within a couple who should have a greater say (the husband, the wife, both equally) in making major household purchases, purchases for daily needs, visits to the wife’s family or relatives, what to do with the money the wife earns, and how many children to have. Men who responded that a wife should have an equal or greater say as her husband in any of the five decisions were considered to have an egalitarian attitude. In contrast, men who said only the husband should have the final say in all of the specified decisions were supposed to have an authoritarian attitude. Men’s justification of wife-beating was assessed through whether they justify wife-beating by answering the following hypothetical circumstances: if she goes out without telling, neglects the house or children, argues with him, refuses to have sex, does not cook food properly, is suspected of being unfaithful, and is disrespectful to in-laws. Responses were dichotomous (yes, no). A value of ‘1’ was assigned to men who justified wife-beating for any of the mentioned reasons, whereas a value of ‘0’ was assigned to men who denied wife-beating for all the reasons.

### Analytical approach

The weighted bivariate percentage of men’s attitudes toward women’s sexual autonomy by predictor variables was estimated using cross-tabulation. The intra-variables differences were checked using Pearson’s chi-square statistics. The multicollinearity test using the variance inflation factor (VIF) showed no evidence of multicollinearity among all the predictors (mean VIF = 1.88, max = 2.95, min = 1.07). Evidence indicates that VIF < 10 is tolerable [38]. Due to the dichotomous nature of the dependent variable, the multivariate logistic regression technique was used to evaluate the net effects of individual, household, and community-level predictors on the outcome variable. The estimated adjusted odds ratio (AOR) with 95% confidence intervals (CI) was used to present the regression results with a p-value set at 0.05. Additionally, an interaction term combining education and place of residence was used to assess the potential interaction effect of these two variables on the outcome variable. The area under receiver operating characteristics (ROC) curve statistics and the Hosmer-Lemeshow test [39] were used to assess the predicted ability and the goodness of fit of the regression model. The Hosmer-Lemeshow goodness of fit test indicated that the regression model developed in this study fits the data well (p = 0.9197). Sample weights were used to restore the sample’s representativeness. All the statistical analyses were performed on weighted data using Stata version 17.0.

## Results

### Men’s attitude toward women’s sexual autonomy

**Fig 1:**
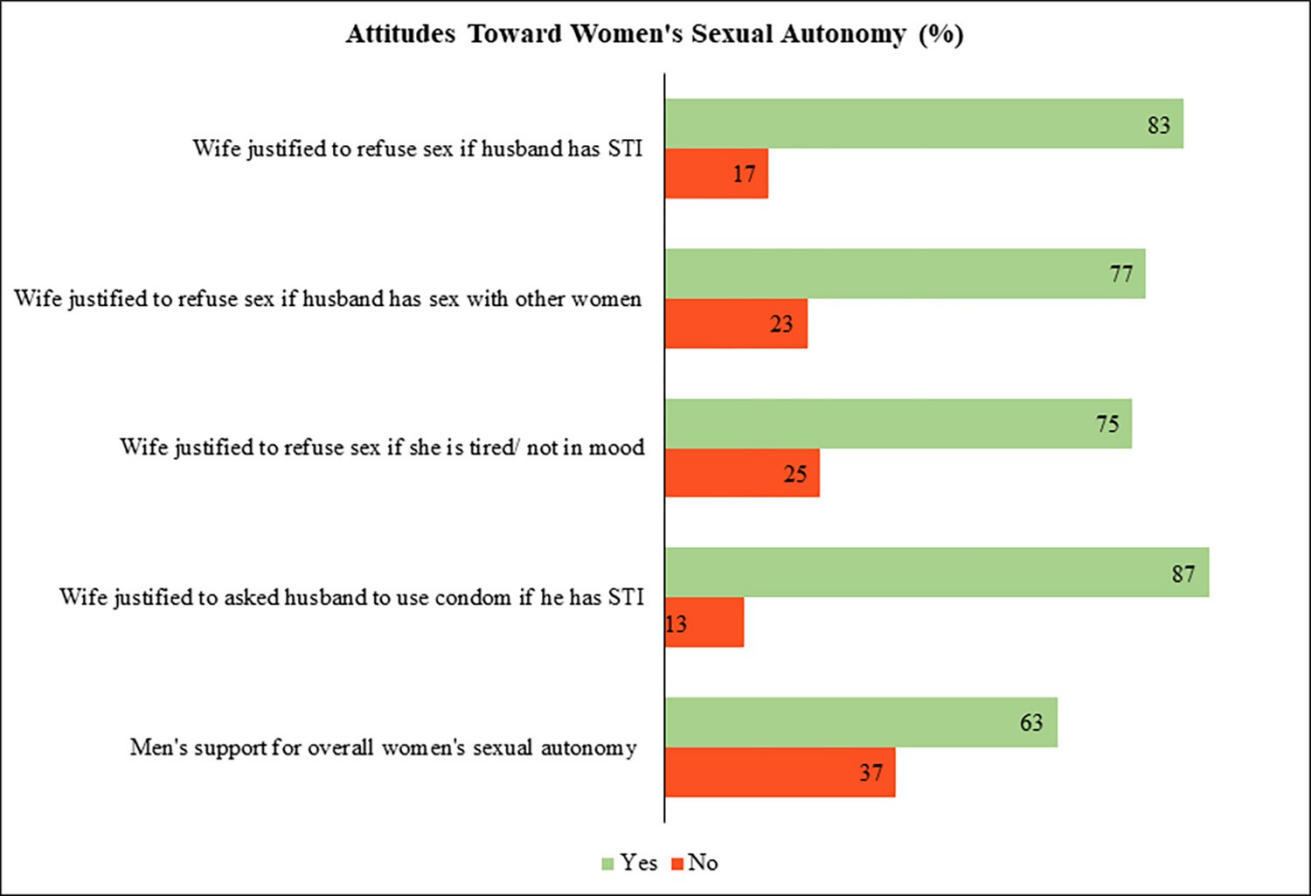
Men’s attitude toward sexual autonomy of women, India, 2019–21.

Nearly two-thirds (63%) of Indian men were in favor of the sexual autonomy of women (Fig 1). 83% of men think that a wife is justified in refusing sex with her husband if she knows her husband has a sexually transmitted disease. 77% of men believe that a wife is justified in refusing sex with her husband if she knows her husband has sex with other women. A wife is justified in refusing sex with her husband if she is tired or not in mood, which was agreed by 75% of men. Again, 87% of men think that the wife is justified in asking the husband to use a condom during intercourse if she knows her husband has a sexually transmitted disease.

### Socio-economic and demographic differentials in men’s attitude toward women’s sexual autonomy

Table 1 presents that a higher percentage of men (68%) with an egalitarian attitude in household decision-making were more in favor of the sexual autonomy of women compared to their authoritarian counterparts (57%). Men who justified wife-beating (54%) were less supportive of the sexual autonomy of women compared to those who did not (70%). Illiterate men (55%) showed less support for the sexual autonomy of women compared to their highly educated (68%) counterparts. Men exposed to mass media (64%) were more in favor of wife’s sexual autonomy than their counterparts. Men who worked in white-collar professions possessed the highest support for the sexual autonomy of women than men who worked in any other occupational categories. Men with a family history of IPV (58%) showed lower support of wife’s sexual autonomy compared to their counterparts (64%). Men’s support for the sexual autonomy of women increased as wealth status increased, with the richest group showing the highest support (68%). Rural men (62%) were less supportive of the sexual autonomy of women than urban men (65%). Men belonging to the southern region (54%) were less supportive of the sexual autonomy of women compared to all other regions.

**Table 1:** Men’s attitude towards sexual autonomy of women by background characteristics, India, 2019–21.

Background Characteristics		Support Women’s Sexual Autonomy		P Value	N
		No	Yes		
**Household Autonomy**				p<0.001	** **
Authoritarian		43.1	56.9		40,686
Egalitarian		32.3	67.7		61,153
**Wife-beating Justified**				p<0.001	
No		30.1	69.9		61,735
Yes		45.8	54.2		40,104
**Age Group**				p<0.001	
15–24		38.9	61.1		31,070
25–34		35.2	64.8		27,652
35–44		36.2	63.9		23,712
45–54		37.9	62.1		19,405
**Education**				p<0.001	
No education		44.8	55.2		12,269
Primary		40.7	59.3		11,710
Secondary		36.4	63.6		60,018
Higher		32.0	68.0		17,842
**Marital Status**				p<0.001	
Never married		38.4	61.6		36,892
Currently married		36.2	63.8		63,377
Others		41.7	58.3		1,570
**Mass Media Exposure**				p<0.001	
No		42.8	57.3		15,253
Yes		36.3	63.8		86,586
**Occupation**				p<0.001	
Not working		39.8	60.2		19,241
White Collar		31.9	68.1		15,187
Agriculture/HH/domst*		39.9	60.1		39,205
Manual labour		34.5	65.5		23,930
Other		37.9	62.1		4,276
**Caste**				p<0.001	
General		35.2	64.8		23,919
SC		37.8	62.2		19,240
ST		37.7	62.3		19,354
OBC		37.9	62.1		39,326
**Religion**				p<0.001	
Hindu		37.1	62.9		77,211
Muslim		38.2	61.8		12,112
Christian		37.7	62.4		7,267
Others		29.3	70.7		5,249
**Family History of IPV**				p<0.001	
No		35.8	64.2		82693
Yes		41.9	58.1		19,146
**Family Structure**				p<0.05	
Nuclear		37.7	62.4		52,289
Non-nuclear		36.4	63.6		49,550
**Wealth Index**				p<0.001	
Poorest		42.4	57.6		19,796
Poorer		38.9	61.1		22,599
Middle		37.8	62.3		21,715
Richer		35.0	65.0		20,209
Richest		32.3	67.7		17,520
**Place of Residence**				p<0.001	
Urban		35.0	65.0		26,420
Rural		38.2	61.8		75,419
**Region**				p<0.001	
North		26.5	73.5		21,134
Central		34.2	65.9		23,242
East		41.6	58.5		15,197
Northeast		34.9	65.1		14,860
West		28.7	71.3		11,588
South		45.9	54.1		15,818
**Total**		37.1	63.0		1,01,839

*HH/domst: Household/domestic services

### Determinants of men’s attitude toward women’s sexual autonomy

Multivariate logistic regression results showed that controlling for other predictors, men with an egalitarian attitude regarding household decision-making were 1.45 (AOR: 1.45; CI: 1.41–1.49) times more likely to be in favor of sexual autonomy of women compared to their authoritarian counterparts (Table 2). Likewise, men who justified wife-beating were 41% less (AOR: 0.59; CI: 0.58–0.61) likely to be in favor of the sexual autonomy of women compared to those who did not justify it. As age increased, the odds of supporting women’s sexual autonomy also increased, with men aged 35 to 44 having the highest odds (AOR: 1.09; CI: 1.03–1.15). Higher levels of education were associated with a greater likelihood (AOR: 1.34; CI:1.20–1.50) of men in favor of the sexual autonomy of women compared to their illiterate counterparts. Additionally, married men were more likely to support sexual autonomy compared to those who were never married. Men exposed to mass media showed 1.17 times more (AOR: 1.17; CI: 1.12–1.21) support for the sexual autonomy of women compared to their non-exposed counterparts. Men in each occupational category showed increased odds of supporting women’s sexual autonomy compared to those in the non-working group. However, men who worked in white-collar jobs (AOR: 1.32; CI:1.26–1.39) and those engaged in manual labor (AOR: 1.27; CI: 1.20–1.33) had slightly higher odds of support for sexual autonomy compared to any other category. Compared to Hindus, Muslims had lower odds (AOR:0.88; CI: 0.84–0.91) of support for sexual autonomy, while Christians had higher odds (AOR: 1.13; CI: 1.04–1.23). Men with a family history of IPV were less likely (AOR: 0.93; CI: 0.90–0.96) to be in favor of the sexual autonomy of women. Higher household wealth was associated with a greater likelihood of men favoring the sexual autonomy of women, with richer men having the highest odds (AOR: 1.17; CI: 1.11–1.23). Men residing in rural areas had lower odds (AOR: 0.87; CI: 0.85–0.90) of support for the sexual autonomy of women compared to their urban counterparts. Men from regions other than the North were less likely to support women’s sexual autonomy. The South region, in particular, stands out with the lowest odds (AOR: 0.63; CI: 0.60–0.67) compared to the North.

**Table 2:** Adjusted odds ratios (AOR) of men’s attitudes toward women’s sexual autonomy, India, 2019–21.

Background Characteristics	Men’s Support for Women’s Sexual Autonomy	
	AOR	95% CI
**Household Autonomy**		
Authoritarian®		
Egalitarian	1.45***	[1.41,1.49]
**Wife-beating Justified**		
No®		
Yes	0.59***	[0.58,0.61]
**Age Group**		
15–24®		
25–34	1.06*	[1.01,1.11]
35–44	1.09**	[1.03,1.15]
45–54	1.07*	[1.01,1.13]
**Education**		
No education®		
Primary	1.19*	[1.04,1.35]
Secondary	1.29***	[1.16,1.43]
Higher	1.34***	[1.20,1.50]
**Marital Status**		
Never married®		
Currently married	1.07**	[1.02,1.12]
Others	1.01	[0.90,1.13]
**Mass Media Exposure**		
No®		
Yes	1.17***	[1.12,1.21]
**Occupation**		
Not working®		
White Collar	1.32***	[1.26,1.39]
Agriculture/HH/domst#	1.05*	[1.01,1.10]
Manual labour	1.27***	[1.20,1.33]
Other	1.15***	[1.07,1.24]
**Caste**		
General®		
SC	0.98	[0.94,1.03]
ST	1	[0.95,1.05]
OBC	1.02	[0.99,1.06]
**Religion**		
Hindu®		
Muslim	0.88***	[0.84,0.91]
Christian	1.13**	[1.04,1.23]
Others	0.99	[0.91,1.08]
**Family History of IPV**		
No®		
Yes	0.93***	[0.90,0.96]
**Family Structure**		
Nuclear®		[1.00,1.00]
Non-nuclear	1.02	[0.99,1.05]
**Wealth Index**		
Poorest®		
Poorer	1.05*	[1.01,1.10]
Middle	1.08**	[1.03,1.13]
Richer	1.14***	[1.08,1.20]
Richest	1.17***	[1.11,1.23]
**Place of Residence**		
Urban®		
Rural	0.87***	[0.85,0.90]
**Region**		
North®		
Central	0.77***	[0.74,0.81]
East	0.79***	[0.75,0.83]
Northeast	0.68***	[0.65,0.72]
West	0.84***	[0.80,0.89]
South	0.63***	[0.60,0.67]
**Interaction of place of residence and education**		
urban × no education	1	[1.00,1.00]
urban × primary	1	[1.00,1.00]
urban × secondary	1	[1.00,1.00]
urban × higher	1	[1.00,1.00]
rural × no education	1	[1.00,1.00]
rural × primary	0.94	[0.82,1.09]
rural × secondary	0.98	[0.88,1.09]
rural × higher	0.99	[0.87,1.12]
N	101839	
Log likelihood	-64448	
The Hosmer-Lemeshow χ^2^ value	0.9197	
Area under ROC curve	0.6281	

®Reference category

95% confidence intervals in brackets

* p<0.05

** p<0.01

*** p<0.001

#HH/domst: Household/domestic services

## Discussion

The study found that nearly two-thirds of Indian men had a favorable attitude toward the sexual autonomy of women. Men’s egalitarian view related to household decision-making, higher educational status, currently married status, exposure to mass media, employment status, and richer household wealth strata were positively associated with their support for the sexual autonomy of women. On the contrary, men who justified wife-beating, were exposed to family violence, resided in rural areas, and belonged to regions other than the North region were negatively associated with support for women’s sexual autonomy.

The present study revealed that men who hold egalitarian views in household decision-making were in favor of the sexual autonomy of women than their authoritarian counterparts. Past studies also found that the controlling attitude of the husband regarding household decisions is negatively associated with the wife’s negotiation of safer sexual practices, such as refusing sex and asking the partner to use a condom [19, 22]. The unequal power distribution within households mostly favors men, granting them authority over the decision-making process, which further leads to control over women’s sexuality, often by subjugating women. Within a dyadic relationship, autonomy in decision-making entails self-determination and freedom from external coercion, which are essential components, especially in the context of engaging in sexual activity [40]. Moreover, women’s involvement in decision-making reduces gender imbalances in the households and minimizes the husband’s controlling attitude, which fosters better decision-making choices regarding unsafe sexual behaviors in risky situations [41].

Another important finding of this study is that men who justified wife-beating were less likely to show a positive attitude about the sexual autonomy of women. An earlier study also found a negative relationship between Indian men’s justification of wife-beating and their support for the sexual autonomy of women [36]. Men’s firm adherence to traditional masculine gender roles encourages them to be dominant over women when it comes to sexual relationships [42]. Moreover, justification for wife-beating by men is associated with higher vulnerability to experiencing sexual violence among women. This directly relates to rationalizing, accepting, and internalizing societal norms that validate such violence [43, 44]. Deep-rooted patriarchal norms urge women should adhere to their husband’s sexual desires and demands without hesitations, and any deviation from these norms, e.g., refusing sex with their husbands or asking their partners to use condoms, may lead to violence perpetration [7].

This study revealed that men’s positive attitude toward the sexual autonomy of women was significantly higher among older age groups compared to younger. This is because older men may better understand intimate relationships than younger men with less experience [45], leading to higher acceptance of the sexual autonomy of the partner. This study found that highly educated men perceived women’s sexual autonomy more favorably compared to their uneducated counterparts. This corroborates earlier research indicating that education plays a role in people’s capacity to adopt gender-egalitarian attitudes and behaviors and empowers them to challenge prevailing cultural norms and values that promote gender inequality [37]. Moreover, highly educated men are more conscious of the sexual rights of women and less likely to adhere to patriarchal sexual norms that restrict women’s sexual freedom and desire [22]. In this study, married men were found to have a positive attitude toward women’s sexual autonomy. This could be explained by the fact that men may better understand dyadic relationships and power dynamics within a marital union [36], leading to greater acceptance of their wife’s sexual rights and bodily autonomy. Men exposed to mass media were also more likely to support women’s sexual autonomy. The use of mass media has been shown to influence safer sexual attitudes, norms, and practices [46]. This study also found that men’s working status was positively associated with the acceptance of the sexual autonomy of women, with men working in white-collar professions more likely to be in favor of women’s sexual autonomy than any other occupational category. A possible justification for this finding is that men engaged in higher employment sectors are mostly highly educated and thus informed about greater egalitarian norms and the sexual rights of partners [47]. Many white-collar professions involve inclusive work environments that encourage critical thinking and progressive social views, fostering greater acceptance of gender equality. Exposure to such environments could lead to a broader acceptance of women’s rights, including sexual autonomy, among working professionals.

This study indicated that Muslim men were significantly less supportive of the sexual autonomy of women compared to their Hindu counterparts. Evidence suggests that Muslims support patriarchal values more than non-Muslims [48]. In Islamic traditions, men are providers for women, and in exchange, women should be submissive toward men and refrain from questioning their authority [47]. This study further revealed that men who witnessed their fathers beating their mothers during childhood were less likely to be in favor of the sexual autonomy of women. Past studies suggest that men who had witnessed or experienced violence during childhood may become perpetrators of wife abuse; as adults, they are more likely to develop attitudes condoning the husband’s right to control or sexually and physically abuse their wives [49, 50].

In this study, men belonging to wealthier strata were found to be more supportive of the sexual autonomy of women. More affluent people are generally exposed to egalitarian gender norms through more years of schooling, and they are likelier to challenge patriarchal norms related to women’s subjugation and sexual exploitation. This study also found that rural men had a less favorable attitude toward women’s sexual autonomy. This could be explained by the fact that urbanization is often related to the rapid erosion of patriarchal values and power relations that define women as subordinate to their husbands and men [51]. Therefore, urban men are more aware of safer sexual practices, sexual needs, and rights of women, whereas, in many villages of India, masculine culture still prevails where men are at a distinct advantage in terms of deployment of power over women [52], entitling them to disapprove of women’s sexual autonomy in intimate relationships. Another finding of this study revealed that compared to the Northern region, men from all other regions of India were less likely to be in favor of the sexual autonomy of women. A past study also mentioned more adherence to male attitudinal norms in Southern India compared to Northern regions [53]. However, this finding is quite contrary to the findings of another study [34], which mentioned the higher prevalence of patriarchal norms in the Northern relative to Southern states. The cause for the inconsistency can be ascribed to the chosen indicators of partial measure of patriarchal norms, which substantially vary in computation and conceptualization [53]. Despite increasing prosperity, levels of female education, and better indicators of human development in the region, men from the southern states of India are still less likely to be in favor of women’s sexual autonomy. Strong adherence to religious and cultural beliefs and traditional gender norms could be a reason for lower support for women’s sexual autonomy among men from this region. An earlier study also observed that men in the southern region justify wife-beating more than their northern counterparts [36]. Though the reasons for this trend amid improved development indicators remain unclear, it’s evident that macroeconomic progress alone does not necessarily bring changes to gender norms [54]. Although this study found significant regional disparities in men’s support for women’s sexual autonomy in India, it couldn’t fully explore the reasons. It recommends more in-depth qualitative surveys across regions to reveal the actual scenario.

This study has a few limitations worth mentioning. First, in the survey, attitudes regarding women’s sexual autonomy were collected from male self-reported responses prone to social desirability biases. Social desirability bias occurs when individuals downplay socially unfavorable attitudes and behaviors while emphasizing attributes viewed more favorably by society [55]. Men may have crafted their answers to reflect what they feel is socially acceptable rather than providing their actual attitudes. This tendency leads to the overreporting of progressive views on women’s sexual autonomy. Second, this study suffers from one methodological constraint in constructing the sexual autonomy variable. There is a chance of missing out on other essential indicators of sexual autonomy due to data unavailability. Third, special attention is necessary while interpreting the opinions of men regarding women’s sexual autonomy. The opinion may not always reflect in the actual behavior. Fourth, the cross-sectional nature of the NFHS dataset prevents us from drawing causal inferences between the studied variables. Moreover, the data does not allow further exploration of cultural variation by regions to understand the regional disparity in attitude to sexual autonomy of women. Despite these shortcomings, to our knowledge, the present study is the first of its kind in India that provides insightful findings about men’s attitudes toward the sexual autonomy of women and thereby contributes to the rarely existing body of literature. The novelty of this study lies in the inclusion of male samples using the most recent nationally representative survey with a robust sampling design, which further enables us to generalize the findings.

## Conclusions

Nearly two out of every three Indian men hold a favorable attitude toward women’s sexual autonomy, indicating diminishing deep-rooted masculine norms related to subjugating women within an intimate relationship. Men’s support for women’s sexual autonomy was positively correlated with their egalitarian views on family decision-making, higher educational attainment, currently married status, media exposure, employment status, and wealthier household strata. Men who justified wife-beating, were exposed to family violence during childhood, resided in rural areas, and belonged to non-north regions had lesser support for women’s sexual autonomy. The findings of this study underscore the implementation of customized policies and targeted interventions aimed at promoting gender egalitarian norms in society through educational campaigns that could foster sexual well-being among individuals and encourage the preservation of women’s sexual rights. Introducing gender equality topics in school curricula can reshape societal norms from an early age. This study further suggests promoting gender egalitarian norms through educational campaigns and broadening existing SRH strategies by including younger, non-literates, unmarried, unemployed, and rural men. Community workshops held by local leaders, grassroots healthcare professionals, and non-governmental organizations can be helpful in promoting gender equality and a favorable attitude toward women’s sexual autonomy in rural areas and regions with lesser support for women’s sexual autonomy.
